# Controlled Fabrication of Wafer-Scale, Flexible Ag-TiO_2_ Nanoparticle–Film Hybrid Surface-Enhanced Raman Scattering Substrates for Sub-Micrometer Plastics Detection

**DOI:** 10.3390/nano14191597

**Published:** 2024-10-03

**Authors:** Fanyi Kong, Chenhua Ji, Gaolei Zhao, Lei Zhang, Zheng Hao, Hu Wang, Jianxun Dai, Huolin Huang, Lujun Pan, Dawei Li

**Affiliations:** 1 School of Optoelectronic Engineering and Instrumentation Science, Dalian University of Technology, Dalian 116024, China; 2 Department of General Medicine, Dalian Municipal Central Hospital Affiliated Dalian University of Technology, Dalian 116033, China; 3 Dalian University of Technology and Belarusian State University Joint Institute, Dalian University of Technology, Dalian 116024, China; 4 School of Physics, Dalian University of Technology, Dalian 116024, China

**Keywords:** surface-enhanced Raman scattering, wafer-scale fabrication, Ag-TiO_2_ hybrids, UV photo-reduction, sub-micrometer plastics detection

## Abstract

As an important trace molecular detection technique, surface-enhanced Raman scattering (SERS) has been extensively investigated, while the realization of simple, low-cost, and controllable fabrication of wafer-scale, flexible SERS-active substrates remains challenging. Here, we report a facile, low-cost strategy for fabricating wafer-scale SERS substrates based on Ag-TiO_2_ nanoparticle–film hybrids by combining dip-coating and UV light array photo-deposition. The results show that a centimeter-scale Ag nanoparticle (AgNP) film (~20 cm × 20 cm) could be uniformly photo-deposited on both non-flexible and flexible TiO_2_ substrates, with a relative standard deviation in particle size of only 5.63%. The large-scale AgNP/TiO_2_ hybrids working as SERS substrates show high sensitivity and good uniformity at both the micron and wafer levels, as evidenced by scanning electron microscopy and Raman measurements. In situ bending and tensile experiments demonstrate that the as-prepared flexible AgNP/TiO_2_ SERS substrate is mechanically robust, exhibiting stable SERS activity even in a large bending state as well as after more than 200 tensile cycles. Moreover, the flexible AgNP/TiO_2_ SERS substrates show excellent performance in detecting sub-micrometer-sized plastics (≤1 μm) and low-concentration organic pollutants on complex surfaces. Overall, this study provides a simple path toward wafer-scale, flexible SERS substrate fabrication, which is a big step for practical applications of the SERS technique.

## 1. Introduction

So far, surface-enhanced Raman scattering (SERS), an important trace molecular detection technique, has been extensively investigated [1,2,3,4]. In particular, it is critical to realize the controlled fabrication of SERS-active substrates for the widespread implementation of the SERS technique. In the past few decades, researchers have sought to fabricate large-scale, uniform, sensitive, and reproducible SERS-active substrates by developing various kinds of methods, mainly including soft lithography [5,6], nanoimprinting lithography [7,8,9], wet chemistry [10,11], vacuum deposition-based techniques [12,13], flame spray pyrolysis [14], and ion-track technology [15,16]. However, most of these approaches are complex, expensive, and not suitable for practical applications. Therefore, realizing simple, low-cost, and controllable fabrication of wafer-scale, flexible SERS-active substrates remains challenging [8,17,18].

A promising approach is to prepare Ag-TiO_2_ nanoparticle–film hybrids [19,20,21]. It has been shown that titanium dioxide (TiO_2_), a metal oxide semiconductor, could perform Raman signal enhancement through a charge transfer mechanism [22]. In addition, TiO_2_ semiconductors decorated with plasmonic metal nanocrystals, such as gold and silver (Ag), could increase SERS activity by utilizing the surface plasmon resonance effect [23]. Recently, Khubezhov et al. prepared Ag/TiO_2_ nanostructured substrates by the thermal oxidation of Ti foil followed by thermal deposition of Ag nanoparticles (AgNPs), whose fabrication process is relatively complicated and expensive [24]. It is also known that the photocatalytic reduction method has the advantages of a simple process, low cost, and no restriction on the size of the substrate. Thus, Ag-TiO_2_ nanoparticle–film hybrids prepared via photocatalytic reduction show great potential in developing large-scale, flexible SERS-active substrates.

In this work, we report a facile, low-cost, three-step strategy for fabricating wafer-scale SERS-active substrates based on Ag-TiO_2_ nanoparticle–film hybrids by combining dip coating and UV light array photo-deposition. The results show that a centimeter-scale AgNP film (~20 cm × 20 cm) could be uniformly photo-deposited on both non-flexible (TiO_2_/glass) and flexible [TiO_2_/polyimide (PI)] substrates, thereby forming an ultra-large-scale AgNP/TiO_2_ hybrid SERS substrate with good uniformity and high sensitivity, as evidenced by scanning electron microscopy (SEM) and Raman measurements. To optimize the SERS performance of the as-prepared wafer-scale AgNP/TiO_2_ substrate, we analyzed the effects of UV light design and UV illumination time on both AgNP film growth and SERS activity. In situ bending and tensile experiments demonstrate that the as-prepared flexible AgNP/TiO_2_ SERS substrate is mechanically robust and exhibits stable SERS activity even in a large bending state as well as after more than 200 tensile cycles. Moreover, the as-prepared flexible AgNP/TiO_2_/PI SERS substrate shows excellent performance in detecting sub-micrometer plastics (≤1 μm) and low-concentration organic pollutants on fruit surfaces. Overall, our strategy provides a simple, low-cost, and controllable path toward wafer-scale, flexible, sensitive SERS substrate fabrication, which is a big step for practical applications of the SERS technique.

## 2. Results and Discussion

**Figure 1:**
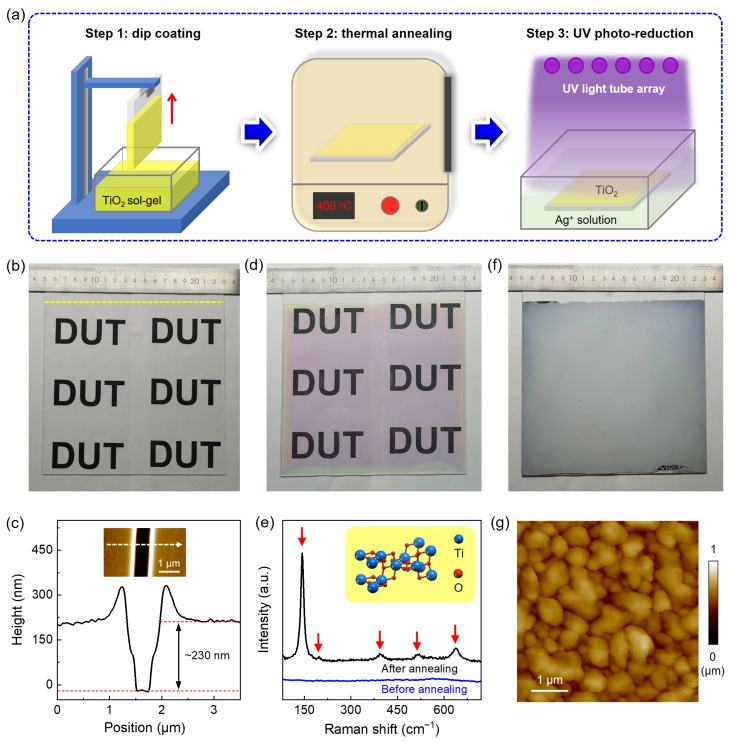
Fabrication and characterization of wafer-scale TiO_2_-catalyzed silver nanoparticle (AgNP) film. (**a**) Schematic of the TiO_2_-catalyzed AgNP film fabrication process, including TiO_2_ thin film deposition via dip coating (Step 1), thermal annealing of the TiO_2_ thin film (Step 2), and UV photo-deposition of the AgNP film (Step 3). (**b**) Photograph of the as-prepared wafer-scale (18 cm × 18 cm) TiO_2_ thin film on a glass substrate, with the corresponding (**c**) AFM image (inset) and height profile along the white dashed line. The yellow dashed line in (**b**) marks the top edge of TiO_2_ thin film. (**d**) Photograph and (**e**) Raman spectrum of the same sample in (**b**) after thermal annealing treatment. The red arrows point to five strong active modes for anatase phase TiO_2_. (**f**) Photograph and (**g**) AFM image of the as-deposited uniform AgNP film on the TiO_2_ surface via UV photo-reduction.

We propose a three-step approach for fabricating wafer-scale Ag-TiO_2_ nanoparticle–film hybrids as SERS-active substrates (Figure 1a), including the formation of TiO_2_ thin film on arbitrary substrate via dip coating (step 1), the thermal annealing of TiO_2_ thin film to improve its crystallinity (Step 2), and the UV photo-deposition of a AgNP film in a Ag ion (Ag^+^) solution (Step 3). To demonstrate the feasibility of this idea, an ultra-large-scale glass with a size up to 18 cm × 18 cm is selected as the substrate. In detail, a transparent TiO_2_ thin film is first deposited onto the glass substrate via dip coating with a lifting rate of 200 mm/min (Figure 1b). Atomic force microscopy (AFM) measurement reveals that the TiO_2_ film is around 230 nm in thickness, which is thick enough for efficient absorption of UV light energy and generation of electron–hole pairs for the photo-deposition of AgNPs (at Stage 3) [19]. Then, the transparent TiO_2_ thin film is annealed at 450 °C for one hour, after which the film changes from colorless to light purple throughout the substrate (Figure 1d), suggesting that phase transition occurs in such a large-scale TiO_2_ thin film. To prove this, Raman measurement is carried out, as shown in Figure 1e. It is obvious that no Raman signal is detected in the TiO_2_ thin film before annealing, indicating an amorphous phase. In contrast, five strong Raman peaks located at 143, 196, 397, 515, and 639 cm^−1^ appear in the annealed TiO_2_ sample, corresponding to the *E*_g_, *E*_g_, *B*_1g_, *A*_1g_, and *E_g_* active modes in the anatase phase [25]. It has been shown that the anatase phase TiO_2_ has much higher photocatalytic activity than the amorphous and other crystal phases like rutile TiO_2_ [26,27,28]. Therefore, the anatase-phase TiO_2_ substrate is chosen for the growth of AgNPs. In stage 3, a UV light tube array with a wavelength of 254 nm and a power density of 18 μW/cm^2^ is used as light source to grow AgNP film on TiO_2_/glass substrate in 3 mM AgNO_3_ solution via photocatalytic reduction. In comparison with that, using a single UV light tube (Figure S1), a highly uniform gray-color AgNP film is grown after irradiation with a UV light tube array (Figure 1f), confirming that wafer-level homogeneous Ag-TiO_2_ nanoparticle–film hybrids are formed (Figure 1g).

**Figure 2:**
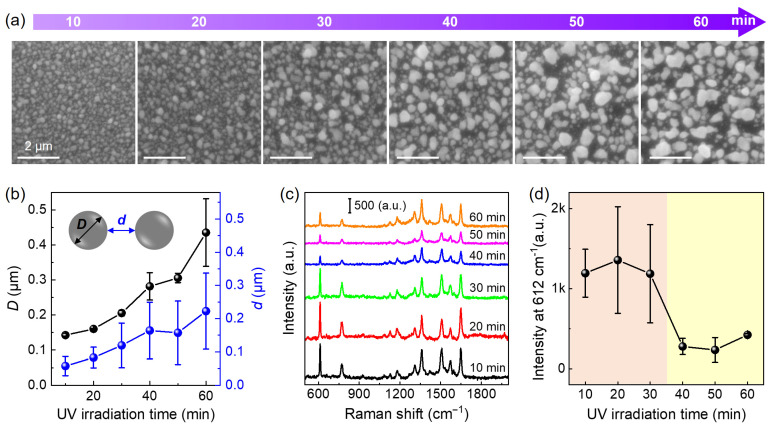
The effect of UV irradiation time on AgNP film growth on the TiO_2_/glass substrate. (**a**) SEM images of AgNP films grown on the TiO_2_/glass substrate with different UV irradiation times. (**b**) The dependence of AgNP size (*D*) and interparticle distance (*d*) on UV irradiation time. Inset: Definition of *D* and *d*. (**c**) SERS spectra of 10^−6^ M R6G taken on as-prepared AgNP/TiO_2_/glass substrates with different UV irradiation times. (**d**) Raman peak intensity at 612 cm^−1^ extracted from (**c**) as a function of UV irradiation time. Color background marks high (*t* < 30 min) and low (*t* > 30 min) SERS activity regions.

Next, we investigate the effect of UV irradiation time on AgNP film growth (Figure 2a and Figure S2), which is critical for Ag-TiO_2_ nanoparticle–film hybrids to serve as an active SERS substrate. Figure 2a shows SEM images of AgNP films grown on the TiO_2_/glass substrate with different UV irradiation times. With a UV irradiation time (*t*) of 10 min, the TiO_2_ film surface produces densely packed AgNPs with an average size of about 143 nm. With the increase in UV irradiation time (10 < *t* ≤ 30 min), part of the AgNPs is increased to a large size; meanwhile, the proportion of large-sized AgNPs is increased. When *t* > 30 min, the proportion of large-sized AgNPs dominates. At *t* = 60 min, the average size of as-grown AgNPs reaches up to 400 nm, which is about 3 times larger than that at *t* = 10 min (Figure 2b). During the photo-deposition of the AgNPs film, an increase in the size of AgNPs with maximal diameters with a simultaneous reduction in the number of small-sized AgNPs is also observed, indicating that TiO_2_-catalyzed AgNP growth is a typical Ostwald ripening process [29], where the unstable small-sized AgNPs tend to dissolve released Ag ions and aggregate into large-sized AgNPs.

To examine the SERS activity of the AgNP/TiO_2_ substrate, R6G is selected as the probe molecule. Figure 2c compares the SERS spectra of 10^−6^ M R6G taken on the as-prepared AgNP/TiO_2_/glass substrates with different UV irradiation times. The fingerprint peaks of R6G molecules at 612 cm^−1^, 773 cm^−1^, 1185 cm^−1^, 1360 cm^−1^, 1508 cm^−1^, 1576 cm^−1^, and 1648 cm^−1^, corresponding to aromatic C-C-C bending, vibrational modes, aromatic C-H bending, C-C stretching, and C=C stretching [30], are clearly detected on all substrates. Figure 2d shows the Raman peak intensity at 612 cm^−1^ extracted from Figure 2c as a function of UV irradiation time. It is obvious that the SERS activity of the AgNP/TiO_2_ substrate with *t* ≤ 30 min is much higher than that with *t* > 30 min. It is known that both nanoparticle size (*D*) and interparticle distance (*d*) play a critical role in SERS activity [31,32,33]. In our case, in the region of 10 ≤ *t* ≤ 30 min, the SERS activity of the AgNP/TiO_2_ substrate is similar, corresponding to the small *d* value and the optimal *D* value (~160 nm), which satisfies the resonance Raman enhancement effect [34]. As *t* > 30 min, the *D* value is further away from the optimal size or λ/4 (λ = 532 nm) meanwhile the *d* value becomes larger (Figure 2b), thus leading to a significant decrease in SERS activity for the AgNP/TiO_2_ substrate. A comparison of SERS efficiency with *D* and *d* values more directly reflects the key role of nanoparticle size and interparticle distance in SERS activity (Figure S3). Moreover, we compare the SERS activity of AgNP/TiO_2_ substrate with bare anatase TiO_2_ substrate, suggesting that both AgNPs and TiO_2_ can perform Raman signal enhancement, while AgNPs play a dominant role (Figure S4).

**Figure 3:**
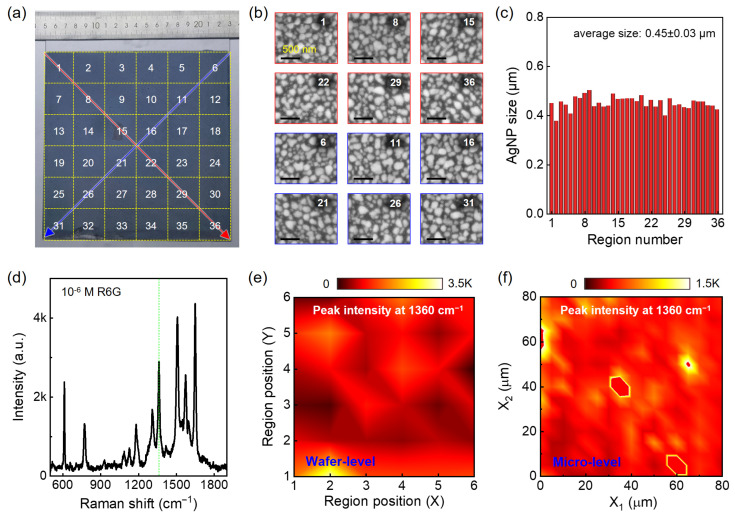
Analyses of structural and SERS properties of the wafer-scale AgNP/TiO_2_/glass substrate. (**a**) Photograph of a wafer-scale AgNP/TiO_2_/glass substrate with 20 min UV irradiation, where the substrate is divided into 36 regions (with a 6 × 6 table) for analysis, with each region labeled using numbers. (**b**) Representative SEM images of twelve regions (**a**) along two diagonal directions (red and blue lines). (**c**) AgNP size distribution in different regions. (**d**) Typical SERS spectrum of 10^−6^ M R6G taken on the AgNP/TiO_2_/glass substrate in (**a**). The green dashed line points to the Raman peak at 1360 cm^−1^. (**e**) Wafer-level and (**f**) micro-level SERS mapping of 10^−6^ M R6G with peak intensity at 1360 cm^−1^. The color bar represents the peak intensity.

Next, we perform structural and SERS characterizations of the wafer-scale AgNP/TiO_2_/glass substrate, which is critical for the practical applications of the SERS technique. Figure 3a shows a photograph of an as-prepared wafer-scale (18 cm × 18 cm) AgNP/TiO_2_/glass substrate. The whole substrate exhibits uniform dark gray color, revealing that AgNP film with highly uniform NP size grows on the TiO_2_ surfaces. To confirm this, the AgNP/TiO_2_/glass substrate is divided into 36 regions, with each region labeled using numbers (Nos. 1-36). Figure 3b shows representative SEM images of twelve regions in Figure 3a along two diagonal directions [red line (Nos. 1, 8, 15, 22, 29, 36) and the blue line (Nos. 6, 11, 16, 21, 26, 31)], all of which show good microscopic homogeneity in NP size and distribution. Furthermore, we statistically analyze the size of AgNPs of each region (Figure 3c), from which the average size of AgNPs is estimated to be 0.45 μm and the relative standard deviation (RSD) is only 5.63%, reflecting the uniformity of AgNPs not only at micron but also at wafer levels.

Next, we examine the SERS activity of the wafer-scale AgNP/TiO_2_/glass substrate. Figure 3d shows a typical SERS spectrum of 10^−6^ M R6G on the AgNP/TiO_2_/glass substrate in Figure 3a. Similar to Figure 2c, seven strong Raman peaks originated from R6G are observed. First, we calculate the enhancement factor (EF) to evaluate the SERS performance of the AgNP/TiO_2_/glass substrate by using the following equation:

(1) $$EF=\left(\frac{I_{SERS}}{c_{SERS}}\right)/\left(\frac{I_{normal}}{c_{normal}}\right),$$

where *I*_SERS_ and *I*_normal_ are the Raman peak signal taken from the AgNP/TiO_2_/glass and SiO_2_/Si substrates, respectively; *c*_SERS_ (10^−6^ M) and *c*_normal_ (10^−1^ M) are the concentrations of the R6G solution used under SERS and normal conditions. The EF value of the AgNP/TiO_2_/glass substrate is estimated to be about 1.3 × 10^6^, comparable to our previously reported results [19,35]. To investigate the SERS uniformity of the AgNP/TiO_2_/glass substrate, we randomly select over ten points from each region in Figure 3a for analysis. Figure 3e shows the mapping of the average Raman peak intensity at 1360 cm^−1^ for 10^−6^ M R6G taken at 36 different region positions, reflecting good uniformity of SERS performance for the AgNP/TiO_2_/glass substrate on the wafer-scale. To evaluate the SERS uniformity at the micro-scale, we also perform R6G SERS mapping at a local area of the substrate, as shown in Figure 3f. As expected, a uniform SERS mapping image is obtained, demonstrating that the wafer-scale Ag/TiO_2_/glass substrate exhibits excellent spot-to-spot reproducibility in SERS activity, consistent with the uniformity in AgNP size (Figure 3c).

**Figure 4:**
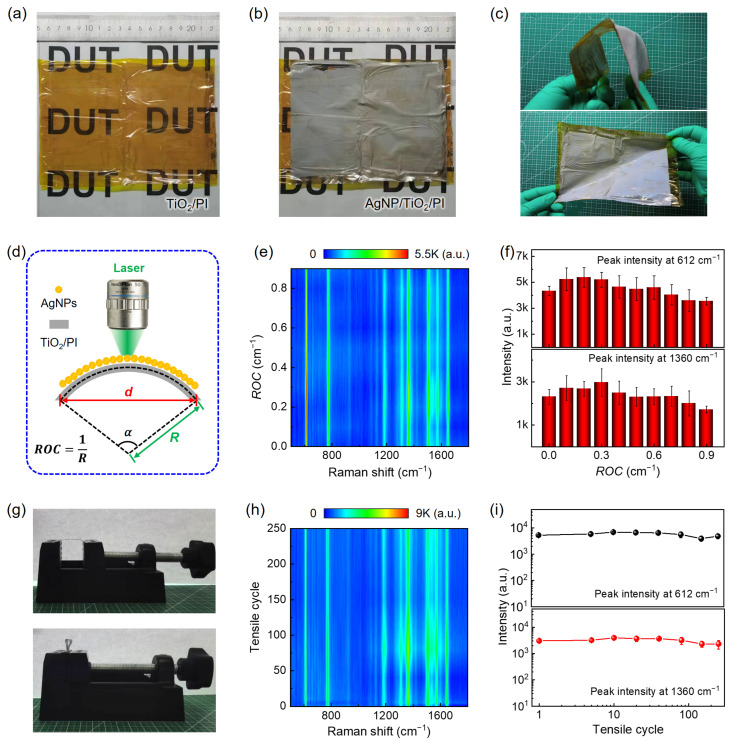
SERS performance of the wafer-scale, flexible AgNP/TiO_2_/PI substrate. (**a**,**b**) Photographs of the as-prepared wafer-scale (**a**) TiO_2_ thin film on the PI substrate and (**b**) AgNP/TiO_2_/PI substrate with 20 min UV irradiation. (**c**) Demonstration of the flexibility of the wafer-scale AgNP/TiO_2_/PI substrate. (**d**) The schematic of the in situ SERS measurement of 10^−6^ M R6G on the AgNP/TiO_2_/PI substrate with different radius of curvature (*ROC*) values, where $ROC=\frac{1}{R}$. (**e**) SERS spectra of 10^−6^ M R6G taken on the AgNP/TiO_2_/PI substrate with the *ROC* varying from 0 to 0.9 cm^−1^. (**f**) The *ROC* dependence of Raman peak intensity at 612 cm^−1^ (top) and 1360 cm^−1^ (bottom) extracted from (**e**). (**g**) Experimental setup for the tensile and bending test. (**h**) SERS spectra of 10^−6^ M R6G taken on the Ag/TiO_2_/PI substrate with different tensile cycles. (**i**) The dependence of Raman peak intensity at 612 cm^−1^ (**top**) and 1360 cm^−1^ (**bottom**) on the tensile cycle.

Based on the above analyses, centimeter-scale AgNP films with good uniformity and high SERS activity are photo-deposited on non-flexible (TiO_2_/glass) substrates. It is expected that this method is also suitable for preparing flexible, wafer-scale Ag-TiO_2_ nanoparticle–film hybrid SERS substrates. To prove this, a polyimide (PI) film is chosen as the flexible substrate. First, we prepare a large-area (15 cm × 12 cm) TiO_2_ thin film on the PI substrate via dip coating (Figure 4a), followed by thermal annealing at 400 °C for one hour. Then, the AgNP film is uniformly grown on the TiO_2_/PI surfaces in 3 mM AgNO_3_ solution under the same UV light tube array irradiation condition (Figure 4b). We find that the as-prepared large-area AgNP/TiO_2_/PI film substrate is not only bendable but also can recover to its original state after bending (Figure 4c).

Next, we investigate the effects of bending strain and tensile cycle on the SERS performance of the AgNP/TiO_2_/PI substrate. To quantitively evaluate the bending strain effect, the flexible AgNP/TiO_2_/PI substrate is cut into a rectangle shape (0.5 cm × 2.5 cm) and affixed to the PDMS film surface for in situ SERS measurement under different radius of curvature (*ROC*) values, where $ROC=\frac{1}{R}$ (Figure S5 and Figure 4d). As shown in Figure 4d, the relationship between the *ROC* and the chord length *d* can be expressed as d=2ROC sin⁡ (360π×ROC). Figure 4e shows the SERS mapping of 10^−6^ M R6G with the *ROC* varying from 0 to 0.9 cm^−1^, where no significant enhancement or attenuation of R6G signals is observed during the bending process. Furthermore, we obtain the *ROC* dependence of Raman peak intensity at 612 cm^−1^ and 1360 cm^−1^ (Figure 4f), where both Raman signals first slightly increase and then gradually decrease with an increasing *ROC*. Compared with the initial state (*ROC* = 0), SERS performance is only decreased by about 15% when the *ROC* reaches 0.9, which can be well explained by slight change in interparticle distance with different *ROC*s. Next, we perform a tensile cycle experiment on the flexible AgNP/TiO_2_/PI SERS substrate, where a tensile cycle consists of once horizontal stretch and once upward bending (Figure 4g). Figure 4h shows the SERS spectra of 10^−6^ M R6G taken on the same AgNP/TiO_2_/PI substrate with different tensile cycles. The dependence of the Raman peak intensity at 612 cm^−1^ and 1360 cm^−1^ extracted from Figure 4h on the tensile cycle is shown in Figure 4i. It can be seen that the SERS signal remains the same order of magnitude with the increase in the number of cycles (even after 250 cycles), demonstrating that the flexible AgNP/TiO_2_/PI SERS substrate is mechanically robust.

**Figure 5:**
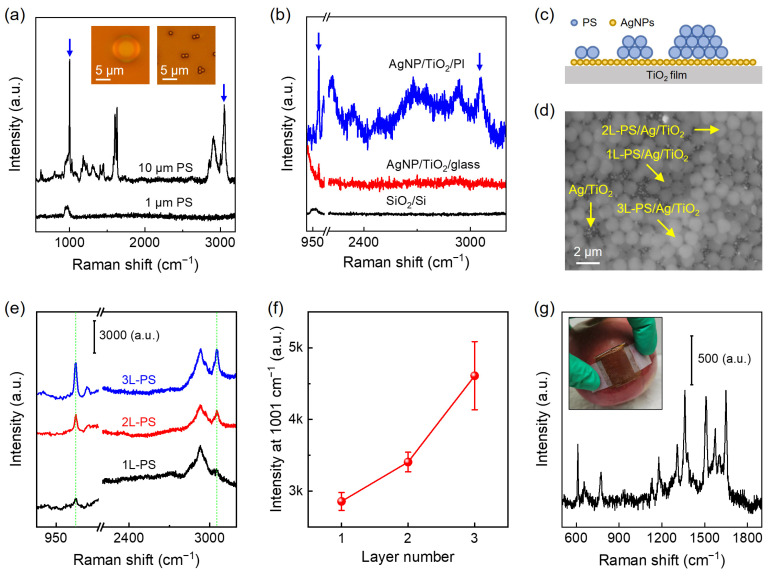
The application of the wafer-scale, flexible AgNP/TiO_2_/PI SERS substrate in sub-micrometer plastics detection. (**a**) Raman spectra of 10 μm and 1 μm sized PS spheres on the SiO_2_/Si substrate, with corresponding optical images (**insets**). (**b**) Raman spectra of 1 μm sized PS spheres on the AgNP/TiO_2_/PI, AgNP/TiO_2_/glass, and SiO_2_/Si substrates. The blue arrows in (**a**,**b**) mark the Raman signal of PS spheres. (**c**) Schematic, (**d**) SEM image, and (**e**) SERS spectra of self-assembled 1L-/2L-/3L-PS sphere arrays on the AgNP/TiO_2_/PI substrate. The yellow symbols in (**d**) point to self-assembled 1L-/2L-/3L-PS sphere arrays, while the green dashed lines in (**e**) mark the Raman signal of PS spheres. (**f**) The dependence of Raman peak intensity at 1001 cm^−1^ extracted from (**e**) on layer number. (**g**) SERS detection of 10^−6^ M R6G on an apple surface (**inset**) using flexible AgNP/TiO_2_/PI substrate.

Finally, we examine the performance of the as-prepared wafer-scale AgNP/TiO_2_ SERS substrate in some practical applications. It has been reported that sub-microplastic (≤1 μm) detection via normal Raman measurement remains challenging [36]. As an example, we compare the Raman spectra of individual polystyrene (PS) spheres with different sizes on the SiO_2_/Si substrate (Figure 5a). The results show that the characteristic Raman peaks at 1001 cm^−1^ (C-H bending mode) and 3055 cm^−1^ (ν4 mode) can be detected from a 10 μm PS sphere [37], while no Raman signal is observed when the size of the PS sphere is reduced to 1 μm. Figure 5b compares the Raman spectra of individual 1 μm PS spheres taken on AgNP/TiO_2_/PI, AgNP/TiO_2_/glass, and SiO_2_/Si substrates under the same conditions, where the Raman signal originated from a 1 μm PS sphere (blue arrows) is successfully detected on both the AgNP/TiO_2_/PI and AgNP/TiO_2_/glass SERS substrates.

During the preparation of PS spheres, we find that self-assembled PS sphere arrays are easily formed (Figure 5c). Figure 5d shows representative SEM images of the 1 μm PS sphere arrays with different layers (L) on the AgNP/TiO_2_ SERS substrate, including 1L-PS, 2L-PS, and 3L-PS. Figure 5e compares the SERS spectra of 1L-/2L-/3L-PS on the AgNP/TiO_2_/PI substrate. We find that the SERS signal of the PS sphere arrays significantly increases with the increment in layer number (Figure 5f), indicating that the micro-plastics with sizes ranging from 1 to 3 μm can be effectively detected via our flexible AgNP/TiO_2_ SERS substrates. On the other hand, the as-prepared wafer-scale, flexible AgNP/TiO_2_ SERS substrate can be easily cut into small pieces and enable the inspection of samples with complex surfaces. As an example, we detect the Raman signal of low-concentration organic pollutants (10^−6^ M R6G) decorated on an apple surface by covering a small piece of Ag/TiO_2_/PI film on top (Figure 5g). It is true that the main Raman peaks originating from R6G are detected. It is expected that the flexible SERS substrate we prepared can be used for easy and quick detection of molecules (such as pesticides) on the surfaces of fruits, vegetables, etc.

## 3. Conclusions

In summary, we realized a controllable fabrication of wafer-scale (~20 cm × 20 cm) SERS-active substrates based on Ag-TiO_2_ nanoparticle–film hybrids via a combined dip-coating and UV light array photo-deposition method. The as-prepared flexible and nonflexible AgNP/TiO_2_ hybrid SERS substrates show good uniformity in both AgNP size and SERS activity ranging from the micron to wafer levels (RSD = 5.63%). In addition, the as-prepared flexible AgNP/TiO_2_ SERS substrate is found to exhibit stable SERS activity even in a large bending state and after hundreds of tensile cycles, strongly supporting its mechanically robust property. Furthermore, we examined and confirmed the SERS sensitivity of the as-prepared wafer-scale, flexible AgNP/TiO_2_ substrate in detecting sub-micrometer plastics (≤1 μm) and low-concentration organic pollutants on fruit surfaces. Overall, our study provides a simple, low-cost path toward wafer-scale, highly sensitive SERS substrate fabrication, which is suitable for mass production and practical applications.

## 4. Methods

Fabrication of wafer-scale AgNP/TiO_2_ hybrid SERS substrates. The fabrication process of the wafer-scale SERS substrates based on AgNP/TiO_2_ heterostructures is as follows. First, a TiO_2_ solution was prepared by a sol-gel method [19,38]. In detail, 50 mL tetrabutyl orthotitanate and 3 mL acetylacetone were mixed and stirred for 10 min (solution A); at the same time, 110 mL alcohol, 1.4 mL deionized water, and 0.2 mL nitric acid were mixed and stirred for 10 min (solution B). Solution B was dropped into solution A during the stirring process. After stirring for 30 min, the TiO_2_ sol solution was formed. Second, a TiO_2_ thin film was deposited onto a wafer-scale (20 cm × 20 cm) glass (or PI) substrate by dip coating with a controlled lifting rate of 200 mm/min. Third, the TiO_2_ thin film was calcined at 400 to 450 °C in a muffle furnace (STM-1-10, SAF Therm) for 1 h. Next, a 3 mM AgNO_3_ solution was prepared by using deionized water as solvent. Finally, the annealed wafer-scale TiO_2_/glass (or TiO_2_/PI) substrate was immersed in the as-prepared AgNO_3_ solution and then irradiated by a UV light array (wavelength: ~254 nm, power density: 18 μW/cm^2^) for a fixed time, after which a large-scale, uniform AgNP film was formed on the TiO_2_/glass (or TiO_2_/PI) substrate.

Structure characterization. The morphology and film thickness were measured in an AFM system (MFP-3D, Oxford) with an AFM tip (Micromesh HQ:NSC14, *k* = 5) working in AC mode. The structure of the as-prepared AgNP films was characterized by SEM (JCM-5000, NeoScope). Raman measurement was conducted in a micro-Raman system (inVia, Renishaw) by focusing a 532 nm laser with a power of ~28.9 μW onto the sample surfaces through a ×50 objective.

SERS activity characterization. To examine the SERS activity of the as-prepared AgNP/TiO_2_ substrate, a 10^−6^ M R6G solution was selected as the probe molecule, which was titrated onto the target surface and dried in air. To examine the capability of micro-plastic detection, micro-sized PS spheres were selected and dispersed onto the AgNP/TiO_2_ SERS substrate.

## Data Availability

The data that support the findings of this study are available on request from the corresponding author.

## Supplementary Materials

The following supporting information can be downloaded at https://www.mdpi.com/article/10.3390/nano14191597/s1: Figure S1. (a) Schematic setup for UV photo-reduction. (b) Photography of a wafer-scale (18 cm × 18 cm) AgNP/TiO_2_/glass substrate prepared using a single UV light tube as an irradiation source. (c–e) SEM images of the (c) top, (d) middle, and (e) bottom regions in (b). Figure S2. Optical images of AgNP/TiO_2_/glass SERS substrates prepared under different UV irradiation times. Figure S3. Raman peak intensity at 612 cm^−1^ as a function of silver (a) nanoparticle size (*D*) and (b) interparticle distance (*d*). The data are extracted from Figure 2b,d in the main text. Figure S4. SERS spectra of 10^−6^ M R6G taken on the AgNP/TiO_2_ (black) and TiO_2_ (red) substrates, and 10^−3^ M R6G taken on the TiO_2_ (blue) substrate. Figure S5. (a) A piece of the AgNP/TiO_2_/PI substrate adhered to a PDMS film. (b) The same sample in (a) under different *ROC* states.

## References

[1] Langer J., Jimenez de Aberasturi D., Aizpurua J., Alvarez-Puebla R.A., Auguié B., Baumberg J.J., Bazan G.C., Bell S.E.J., Boisen A., Brolo A.G. (2020). Present and Future of Surface-Enhanced Raman Scattering. ACS Nano.

[2] Bell S.E.J., Charron G., Cortés E., Kneipp J., de la Chapelle M.L., Langer J., Procházka M., Tran V., Schlücker S. (2020). Towards Reliable and Quantitative Surface-Enhanced Raman Scattering (SERS): From Key Parameters to Good Analytical Practice. Angew. Chem. Int. Ed..

[3] Liu S., Li S., Gao S. (2024). High-Throughput Broad-Spectrum Analysis of Tetracyclines via Surface-Enhanced Raman Spectroscopy Imaging Technology. Chem. Eng. J..

[4] Wang D., Shi F., Jose J., Hu Y., Zhang C., Zhu A., Grzeschik R., Schlücker S., Xie W. (2022). In Situ Monitoring of Palladium-Catalyzed Chemical Reactions by Nanogap-Enhanced Raman Scattering using Single Pd Cube Dimers. J. Am. Chem. Soc..

[5] Cerf A., Molnár G., Vieu C. (2009). Novel Approach for the Assembly of Highly Efficient SERS Substrates. ACS Appl. Mater. Interfaces.

[6] Liu G.L., Lee L.P. (2005). Nanowell Surface Enhanced Raman Scattering Arrays Fabricated by Soft-Lithography for Label-Free Biomolecular Detections in Integrated Microfluidics. Appl. Phys. Lett..

[7] Das A., Pant U., Cao C., Moirangthem R.S., Kamble H.B. (2023). Fabrication of plasmonic nanopyramidal array as flexible SERS substrate for biosensing application. Nano Res..

[8] Suresh V., Ding L., Chew A.B., Yap F.L. (2018). Fabrication of Large-Area Flexible SERS Substrates by Nanoimprint Lithography. ACS Appl. Nano Mater..

[9] Ding T., Sigle D.O., Herrmann L.O., Wolverson D., Baumberg J.J. (2014). Nanoimprint Lithography of Al Nanovoids for Deep-UV SERS. ACS Appl. Mater. Interfaces.

[10] Wang L., Li H., Tian J., Sun X. (2010). Monodisperse, Micrometer-Scale, Highly Crystalline, Nanotextured Ag Dendrites: Rapid, Large-Scale, Wet-Chemical Synthesis and Their Application as SERS Substrates. ACS Appl. Mater. Interfaces.

[11] Lee C.-W., Chia Z.C., Hsieh Y.-T., Tsai H.-C., Tai Y., Yu T.-T., Huang C.-C. (2021). A Facile Wet-Chemistry Approach to Engineer an Au-Based SERS Substrate and Enhance Sensitivity Down to ppb-Level Detection. Nanoscale.

[12] Xie Z., Zhao F., Zou S., Zhu F., Zhang Z., Wang W. (2021). TiO_2_ Nanorod Arrays Decorated with Au Nanoparticles as Sensitive and Recyclable SERS Substrates. J. Alloys Compd..

[13] Wang Y.-C., DuChene J.S., Huo F., Wei W.D. (2014). An in situ Approach for Facile Fabrication of Robust and Scalable SERS Substrates. Nanoscale.

[14] Li H., Dumont E., Slipets R., Thersleff T., Boisen A., Sotiriou G.A. (2023). Democratizing Robust SERS Nano-Sensors for Food Safety Diagnostics. Chem. Eng. J..

[15] Yakimchuk D.V., Bundyukova V.D., Ustarroz J., Terryn H., Baert K., Kozlovskiy A.L., Zdorovets M.V., Khubezhov S.A., Trukhanov A.V., Trukhanov S.V. (2020). Morphology and Microstructure Evolution of Gold Nanostructures in the Limited Volume Porous Matrices. Sensors.

[16] Yakimchuk D.V., Prigodich U.V., Demyanov S.E., Ustarroz J., Terryn H., Baert K., Khubezhov S.A., Tishkevich D.I., Trukhanov A.V., Sivakov V. (2022). Growth Mechanism Study of Silver Nanostructures in a Limited Volume. Mater. Chem. Phys..

[17] Li Z., Huang X., Lu G. (2020). Recent Developments of Flexible and Transparent SERS Substrates. J. Mater. Chem. C.

[18] Liu H., He Y., Cao K. (2021). Flexible Surface-Enhanced Raman Scattering Substrates: A Review on Constructions, Applications, and Challenges. Adv. Mater. Interfaces.

[19] Li D., Pan L., Li S., Liu K., Wu S., Peng W. (2013). Controlled Preparation of Uniform TiO_2_-Catalyzed Silver Nanoparticle Films for Surface-Enhanced Raman Scattering. J. Phys. Chem. C.

[20] Suganami Y., Oshikiri T., Mitomo H., Sasaki K., Liu Y.-E., Shi X., Matsuo Y., Ijiro K., Misawa H. (2024). Spatially Uniform and Quantitative Surface-Enhanced Raman Scattering under Modal Ultrastrong Coupling Beyond Nanostructure Homogeneity Limits. ACS Nano.

[21] Zalduendo M.M., Oestreicher V., Langer J., Liz-Marzán L.M., Angelomé P.C. (2020). Monitoring Chemical Reactions with SERS-Active Ag-Loaded Mesoporous TiO_2_ Films. Anal. Chem..

[22] Yang L., Qin X., Jiang X., Gong M., Yin D., Zhang Y., Zhao B. (2015). SERS Investigation of Ciprofloxacin Drug Molecules on TiO_2_ Nanoparticles. Phys. Chem. Chem. Phys..

[23] Lu J., Yang J., Singh S.C., Zhan Z., Yu Z., Xin W., Huang T., Guo C. (2019). Hierarchical Micro/nanostructured TiO_2_/Ag Substrates Based on Femtosecond Laser Structuring: A Facile Route for Enhanced SERS Performance and Location Predictability. Appl. Surf. Sci..

[24] Khubezhov S.A., Ponkratova E.Y., Kuzmichev A.M., Maleeva K.A., Larin A.O., Karsakova M.E., Yakimchuk D.V., Zyuzin M.V., Makarov S.V., Zuev D.A. (2024). Fast and Scalable Fabrication of Ag/TiO_2_ Nanostructured Substrates for Enhanced Plasmonic Sensing and Photocatalytic Applications. Appl. Surf. Sci..

[25] Orendorz A., Brodyanski A., Lösch J., Bai L.H., Chen Z.H., Le Y.K., Ziegler C., Gnaser H. (2007). Phase Transformation and Particle Growth in Nanocrystalline Anatase TiO_2_ Films Analyzed by X-ray Diffraction and Raman Spectroscopy. Surf. Sci..

[26] Ovenstone J., Yanagisawa K. (1999). Effect of Hydrothermal Treatment of Amorphous Titania on the Phase Change from Anatase to Rutile during Calcination. Chem. Mater..

[27] Ohtani B., Ogawa Y., Nishimoto S.-i. (1997). Photocatalytic Activity of Amorphous–Anatase Mixture of Titanium(IV) Oxide Particles Suspended in Aqueous Solutions. J. Phys. Chem. B.

[28] Zhang J., Zhou P., Liu J., Yu J. (2014). New Understanding of the Difference of Photocatalytic Activity Among Anatase, Rutile and Brookite TiO_2_. Phys. Chem. Chem. Phys..

[29] Wang F., Richards V.N., Shields S.P., Buhro W.E. (2014). Kinetics and Mechanisms of Aggregative Nanocrystal Growth. Chem. Mater..

[30] Wang P., Liang O., Zhang W., Schroeder T., Xie Y.-H. (2013). Ultra-Sensitive Graphene-Plasmonic Hybrid Platform for Label-Free Detection. Adv. Mater..

[31] Njoki P.N., Lim I.I.S., Mott D., Park H.-Y., Khan B., Mishra S., Sujakumar R., Luo J., Zhong C.-J. (2007). Size Correlation of Optical and Spectroscopic Properties for Gold Nanoparticles. J. Phys. Chem. C.

[32] Bell S.E.J., McCourt M.R. (2009). SERS Enhancement by Aggregated Au Colloids: Effect of Particle Size. Phys. Chem. Chem. Phys..

[33] Serrano A., Rodríguez de la Fuente O., García M.A. (2010). Extended and Localized Surface Plasmons in Annealed Au Films on Glass Substrates. J. Appl. Phys..

[34] Zhao Y., Zhang Y.-J., Meng J.-H., Chen S., Panneerselvam R., Li C.-Y., Jamali S.B., Li X., Yang Z.-L., Li J.-F. (2016). A Facile Method for the Synthesis of Large-Size Ag Nanoparticles as Efficient SERS Substrates. J. Raman Spectrosc..

[35] Li S., Tao Q., Li D.-W., Liu K., Zhang Q.-Y. (2014). Photocatalytic Growth and Plasmonic Properties of Ag Nanoparticles on TiO_2_ Films. J. Mater. Res..

[36] Xu G., Cheng H., Jones R., Feng Y., Gong K., Li K., Fang X., Tahir M.A., Valev V.K., Zhang L. (2020). Surface-Enhanced Raman Spectroscopy Facilitates the Detection of Microplastics <1 μm in the Environment. Environ. Sci. Technol..

[37] Sears W.M., Hunt J.L., Stevens J.R. (1982). Raman Spectra at Low Temperatures and Depolarization Ratios for Styrene and Polystyrene. J. Chem. Phys..

[38] Li D., Pan L., Wu S., Li S. (2013). An Active Surface Enhanced Raman Scattering Substrate Using Carbon Nanocoils. J. Mater. Res..

