# Supplementary material for: Controlled Fabrication of Wafer-Scale, Flexible Ag-TiO2 Nanoparticle–Film Hybrid Surface-Enhanced Raman Scattering Substrates for Sub-Micrometer Plastics Detection
Source: Nanomaterials (Basel). 2024 Oct 3;14(19):1597. doi: 10.3390/nano14191597 (PMC11477886; doi:10.3390/nano14191597)
Supplement: Supplementary file 1 [file nanomaterials-14-01597-s001.zip › nanomaterials-3212997-supplementary.pdf]

Supporting Information for

Controlled Fabrication of Wafer-Scale, Flexible Ag-TiO_2 Nanoparticle–Film Hybrid Surface-Enhanced Raman Scattering Substrates for Sub-micrometer Plastics Detection

Fanyi Kong [1], Chenhua Ji [2], Gaolei Zhao [1], Lei Zhang [1], Zheng Hao [3], Hu Wang [1], Jianxun Dai [1], Huolin Huang [1], Lujun Pan [4] and Dawei Li [1,3,*]

[1] School of Optoelectronic Engineering and Instrumentation Science, Dalian University of Technology, Dalian 116024, China
[2] Department of General Medicine, Dalian Municipal Central Hospital Affiliated Dalian University of Technology, Dalian 116033, China
[3] Dalian University of Technology and Belarusian State University Joint Institute, Dalian University of Technology, Dalian 116024, China
[4] School of Physics, Dalian University of Technology, Dalian 116024, China
* Correspondence: dwli@dlut.edu.cn

1. Photo-deposition of AgNP film on TiO_2/Glass substrate with single UV light tube

2. The effect of UV irradiation time on AgNP film growth

3. The dependence of SERS efficiency on nanoparticle size and interparticle distance

4. Comparison of SERS activity of AgNP/TiO_2 and TiO_2 substrates

5. Flexible AgNP/TiO_2/PI substrate at different *ROC* states

1. Photo-deposition of a AgNP film on a TiO_2/glass substrate with a single UV light tube.

Figure S1a shows the schematic setup for silver nanoparticle (AgNP) film photo-deposition on a TiO_2/glass substrate under single UV light tube irradiation. Figure S1b shows a photography of a wafer-scale Ag-TiO_2 nanoparticle–film hybrid substrate prepared with 60 min single UV light tube irradiation. It can be seen that the color of the AgNP/TiO_2/glass SERS substrate is not uniform. The middle region has the lowest transmittance, while the top and bottom regions have the highest transmittance. It is indicated that dense and large AgNP growth occurs in the middle region, and AgNPs with small sizes and large interparticle distances are grown in the top or bottom regions, as evidenced by scanning electron microscopy measurements (Figure S1c-S1e). The phenomenon observed above is attributed to the uneven irradiation by the single UV light tube. Therefore, the homogeneity in UV light irradiation determines the uniformity in the as-grown AgNP film.

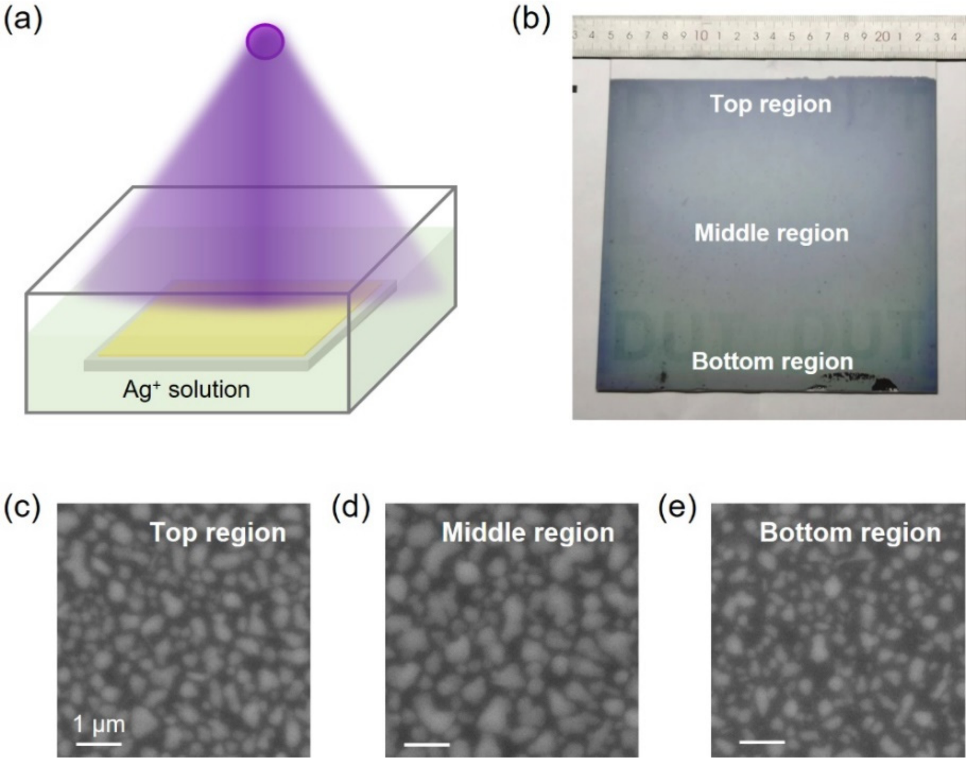

Figure S1. (a) Schematic setup for UV photo-reduction. (b) Photography of a wafer-scale (18 cm × 18 cm) AgNP/TiO_2/glass substrate prepared using a single UV light tube as the irradiation source. (c-e) SEM images of the (c) top, (d) middle and (e) bottom regions in (b).

2. The effect of UV irradiation time on AgNP film growth.

Figure S2 exhibits optical images of the AgNP/TiO_2/glass SERS substrates under UV light tube array irradiation for 10 to 60 min. As the irradiation time increases, the brightness of the sample also increases, which is attributed to the increased density and size of AgNPs, as confirmed by SEM measurement (Figure 2a in the main text).

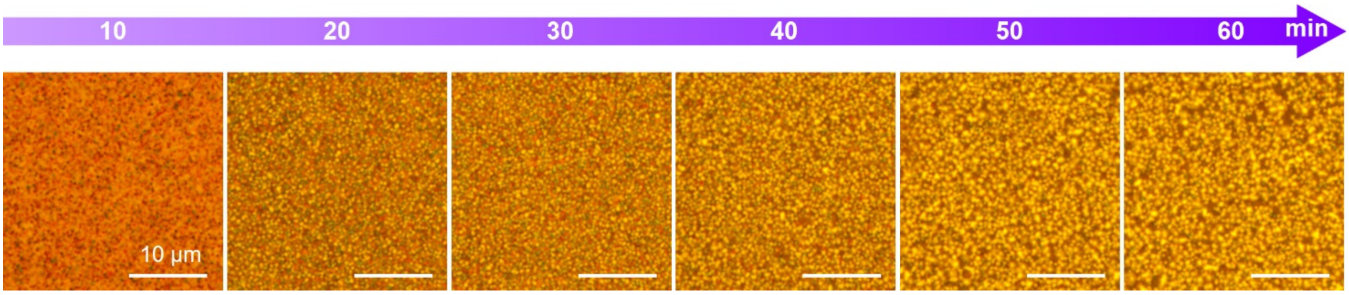

Figure S2. Optical images of the AgNP/TiO_2/glass SERS substrates prepared under different UV irradiation times.

3. The dependence of SERS efficiency on nanoparticle size and interparticle distance.

Figure 2d in the main text shows the Raman peak intensity at 612 cm^{-1} extracted from Figure 2c as a function of UV irradiation time. Moreover, we have compared the Raman intensity at 612 cm^{-1} as a function of silver nanoparticle size (*D*) (Figure S3a) and interparticle distance (*d*) (Figure S3d). Consistent with the analyses in Figure 2 in the main text, the AgNPs/TiO_2 substrate exhibits higher SERS activity with small *D* and *d* values than that with large *D* and *d* values, which can be well explained by resonance Raman enhancement and "hot spot" effects.

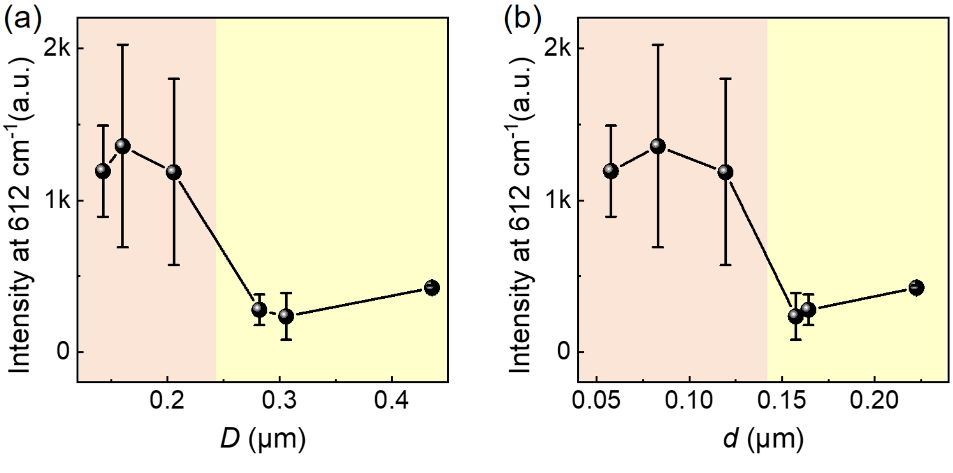

Figure S3. Raman peak intensity at 612 cm^{-1} as a function of silver (a) nanoparticle size (*D*) and (b) interparticle distance (*d*). The data are extracted from Figure 2b,d in the main text.

4. Comparison of SERS activity of AgNP/TiO_2 and TiO_2 substrates.

Figure S4 compares the SERS spectra of 10^{-6} M R6G taken on AgNP/TiO_2 (black) and TiO_2 (red) substrates, suggesting that AgNPs play a dominate role in signal enhancement, which is attributed to surface plasmon resonance effect. In addition, we have measured the SERS spectra

of 10^{-3} M R6G on bare TiO_2 substrate, where weak Raman signal originated from R6G is detected. It indicates that anatase TiO_2 can also perform Raman signal enhancement, which can be well explained by charge transfer mechanism.

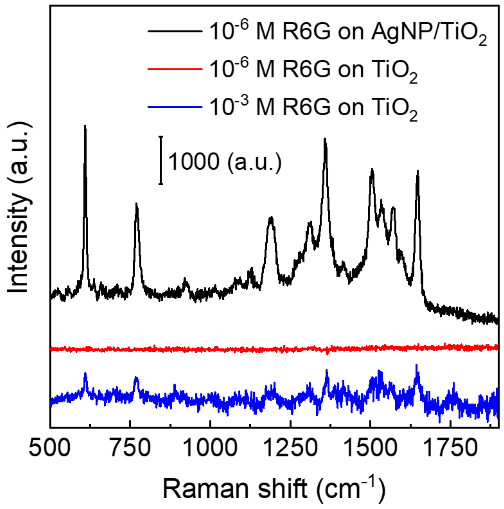

Figure S4. SERS spectra of 10^{-6} M R6G taken on AgNP/TiO_2 (black) and TiO_2 (red) substrates, and 10^{-3} M R6G taken on TiO_2 (blue) substrate.

5. The flexible AgNP/TiO_2/PI substrate at different *ROC* states.

Figure S3a shows a photography of a piece of the AgNP/TiO_2/PI SERS substrate adhered to a PDMS film with a chord length of *d*. We could realize different bending strains on the AgNP/TiO_2/PI SERS substrate by keeping the PDMS film at different chord lengths *d* (Figure 4d in the main text). Figure S3b shows the AgNP/TiO_2/PI SERS substrate on the PDMS film with a radius of curvature (*ROC*) ranging from 0 to 0.9.

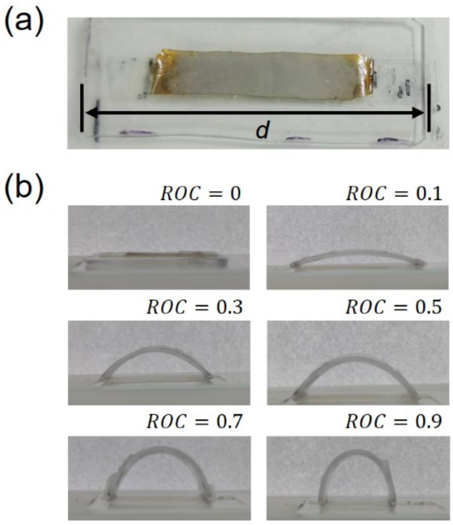

Figure S5. (a) A piece of the AgNP/TiO_2/PI substrate adhered to a PDMS film. (b) The same sample in (a) under different *ROC* states.
